# The combination operation of grouping and ensemble coding for structured biological motion crowds in working memory

**DOI:** 10.1186/s41235-024-00574-6

**Published:** 2024-07-10

**Authors:** Wei Chen, Shujuan Ye, Xin Yan, Xiaowei Ding

**Affiliations:** Present Address: Department of Psychology, Sun Yat-Sen University, Guangzhou, China

**Keywords:** Biological motion, Working memory, Grouping, Ensemble encoding, Gestalt principles

## Abstract

Massive studies have explored biological motion (BM) crowds processing for their remarkable social significance, primarily focused on uniformly distributed ones. However, real-world BM crowds often exhibit hierarchical structures rather than uniform arrangements. How such structured BM crowds are processed remains a subject of inquiry. This study investigates the representation of structured BM crowds in working memory (WM), recognizing the pivotal role WM plays in our social interactions involving BM. We propose the group-based ensemble hypothesis and test it through a member identification task. Participants were required to discern whether a presented BM belonged to a prior memory display of eight BM, each with distinct walking directions. Drawing on prominent Gestalt principles as organizational cues, we constructed structured groups within BM crowds by applying proximity and similarity cues in Experiments 1 and 2, respectively. In Experiment 3, we deliberately weakened the visibility of stimuli structures by increasing the similarity between subsets, probing the robustness of results. Consistently, our findings indicate that BM aligned with the mean direction of the subsets was more likely to be recognized as part of the memory stimuli. This suggests that WM inherently organizes structured BM crowds into separate ensembles based on organizational cues. In essence, our results illuminate the simultaneous operation of grouping and ensemble encoding mechanisms for BM crowds within WM.

**Supplementary Information:**

The online version contains supplementary material available at 10.1186/s41235-024-00574-6.

As social creatures, our ability to perceive and comprehend the motion of biological entities in crowds is of paramount importance. The social information available in crowds of biological motion (BM) not only benefits interpersonal interactions, but also provides clues about immediate predation risk and food resources (Galef & Giraldeau, 2001; Griffin, 2004). Consequently, crowds processing has intrigued vast investigations, including perceptions of emotions and intentions conveyed by BM crowds (e.g., Brunyé et al., 2014; Helbing et al., 2000; Moussaïd et al., 2016), the working memory capacity dedicated to BM crowds (e.g., Ding et al., 2017), and crowd attention (e.g., Gallup et al., 2012; Ristic & Capozzi, 2022; Sweeny & Whitney, 2014), wherein the majority concerned uniformly distributed BM or BM crowds with high homogeneity. However, in reality, BM crowds frequently exhibit clustering structures rather than uniform arrangements. For instance, consider a basketball game where players inherently belong to two distinct teams, or a group seated on a lawn, often organized in pairs or trios rather than forming a unified entity. Despite these observations, the cognitive mechanisms underlying the processing of structured BM crowds remain insufficiently understood.

To address this gap, we embarked on an exploration of structured BM crowds processing by examining their storage in working memory (WM), recognizing the crucial role of WM storage for BM in our daily social life. Numerous studies have examined the WM aspects related to BM, including BM's WM capacity (Shen et al., 2014; Smyth & Pendleton, 1989; Smyth et al., 1988; Wood, 2007, 2011), the binding between BM and other features (Ding et al., 2015; Lu et al., 2019; Wood, 2008, 2010), and the neural mechanisms underlying BM's representations in WM (Cai et al., 2018; Gao et al., 2015; Lu et al., 2016). However, these investigations have primarily centered on WM pertaining to individual BM or unstructured BM crowds, thereby failing to address the specificities of structured BM crowds. The present study aims to elucidate how structured BM crowds are stored in WM.

Ensemble coding may provide an efficient solution allowing WM to circumvent capacity limitations and simultaneously process substantial amount of information, whereby the statistical structure of BM crowds is rapidly and accurately extracted and maintained in WM (e.g., mean and variance; Alvarez, 2011; Michael et al., 2014; Whitney & Yamanashi Leib, 2018). This mechanism has undergone extensive exploration in relation to fundamental physical attributes (Ariely, 2001; Baek & Chong, 2020; Parkes et al., 2001; Webster et al., 2014), social traits (Elias et al., 2017; Lee & Chong, 2021; Marini et al., 2023; Sweeny et al., 2013), and even semantic categories (Khayat & Hochstein, 2019; Khayat et al., 2021) in perception. Within the realm of WM, this phenomenon also garners support from both behavioral findings (Brady & Alvarez, 2015; Schurgin & Brady, 2019; Son & Chong, 2023; Son et al., 2019; Utochkin & Brady, 2020) and neural evidence (Oh et al., 2019). Furthermore, this encoding process within WM seems to operate in an automatic fashion, as suggested by implicit member identification tasks. For instance, participants tasked with judging whether a probe belonged to a prior memory display showed a stronger inclination toward endorsing the probe as present in the memory display when it closely approximated the mean value of all stimuli (Khayat & Hochstein, 2018, 2019; Khayat et al., 2021; Oh et al., 2019). Drawing inspiration from these outcomes, which reaffirm the widespread and inherent nature of ensemble coding in WM, a practical and effective approach to representing structured BM crowds involves retaining their summary statistics, such as means.

However, considering the profound impact of the stimuli structure on WM representation, a seemingly more reasonable hypothesis is that structured BM crowds are stored within WM as discrete ensembles, guided by their organizational cues. Extensive research has consistently revealed that information stored in WM inherently follows a hierarchical organization based on its structural attributes. Within this hierarchy, Gestalt grouping cues hold a pivotal role, encompassing factors like proximity, connectedness, and shared spatial region (e.g., Brady et al., 2011; Corbett, 2017; Gao et al., 2016a, 2016b; Peterson & Berryhill, 2013; Peterson et al., 2015; Son et al., 2019). From the computational level, ensemble representations, considered as pooling population responses, could naturally exhibit similarity-based clustering and segmentation effects (Im et al., 2021; Utochkin, 2015; Utochkin et al., 2024; Treue et al., 2000). Furthermore, within the domain of studies on memorizing BM, research has indicated that social relationships within BM can enhance WM performance, implying the extraction of crowd structure (Ding et al., 2017; Vestner et al., 2019, 2022). In light of these findings, we posit that the structural composition of BM crowds significantly influences their WM representations. Taken together, we propose a group-based ensemble hypothesis to tackle the storage of structured BM crowds in WM, which assumes that BM crowds undergo an automatic organization into distinct subsets and are maintained as separate ensembles within WM.

The direction of walking stands as a fundamental attribute of BM. To test the group-based ensemble hypothesis, the current study employed point light displays (PLDs) to depict walking, a method demonstrated to effectively probe the cognitive processing of human actions (e.g., Abernethy et al., 2001; Blake & Shiffrar, 2007; Johansson, 1973). Adopting the member identification task mentioned earlier (Oh et al., 2019), participants were tasked with determining whether a presented stimulus belonged to a previous memory display. This paradigm deliberately avoided explicit cues for participants to report the mean value of all stimuli, thereby offering a substantial advantage in implicitly detecting the automatic formation of ensemble representations. If a BM representation characterized by a specific walking direction is held in WM, it would be more prone to recognition as part of the memory stimuli. Thus, under the premise of the group-based ensemble hypothesis, the means of subsets would emerge as the most probable attributes to be perceived as components of the memory display (Fig. 1).

**Fig. 1:**
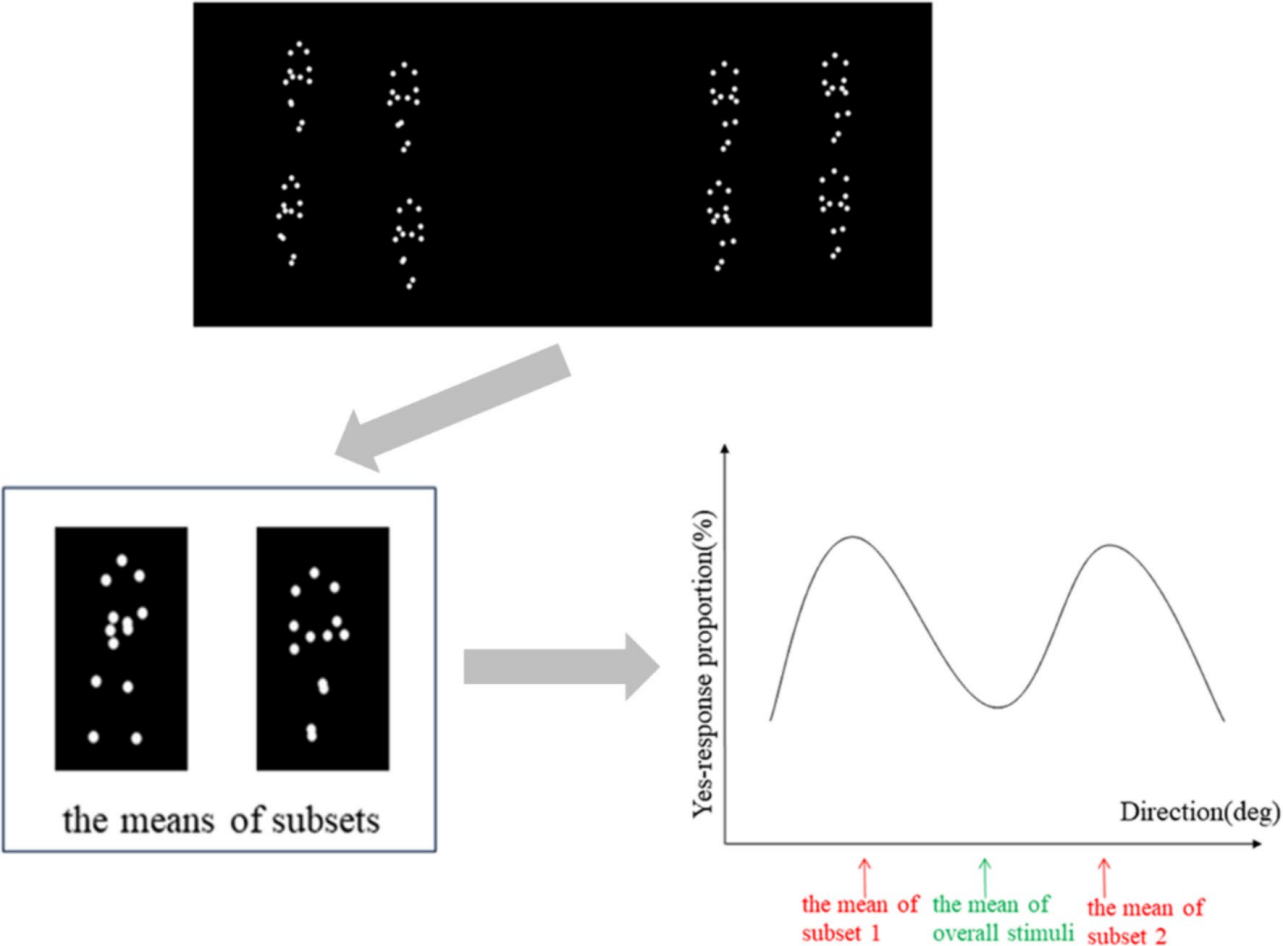
Predictions of the group-based ensemble hypothesis. The group-based ensemble hypothesis predicts the highest proportion of ‘yes’ response to the BM featuring the mean direction of the subsets of memory stimuli

The underlying physical structure is a fundamental hallmark of group-based BM, typically adhering to Gestalt principles. Consequently, our study set out to manipulate two key Gestalt principles to emulate structured BM crowds: proximity (Experiment 1) and similarity (Experiments 2–3). In Experiment 1, eight BM exhibiting diverse directions were grouped based on their proximity, resulting in an equitable distribution on both sides of the screen. Experiments 2 and 3 positioned eight BM uniformly along an unseen circle centered on the screen. To achieve similarity-driven grouping, we positioned these stimuli in tight clusters around two distinct directions, with four stimuli aligned with each direction. The sole distinction between Experiments 2 and 3 lay in the composition of BM' walking directions. In Experiment 2, half of the stimuli walked to the left, while the remainder walked to the right, while in Experiment 3, all BM were directed either leftward or rightward. This strategic choice diminished the visibility of stimuli structures, enabling an assessment of result robustness.

## Experiment 1: WM representations for BM crowds structured by proximity

### Methods

#### Participants

Twenty volunteers from Sun Yat-sen University participated in this experiment for payment. Two participants were subsequently excluded due to abnormal response patterns, characterized by unusually high proportions of 'yes' responses across all conditions. The remaining 18 participants (11 males and 7 females, M = 19.89 years old) were all right-handed and reported normal or corrected-to-normal visual acuity.

The sample size was determined a priori based on PANGEA (Westfall, 2016). Based on the results of previous studies (Oh et al., 2019; the responses of ‘yes’ at the mean orientation vs. the proportion combined across all memory orientations in the varied orientation condition of Experiment 1; *t*(19) = 3.74, *p* = 0.001), which used the similar design with ours, we calculated the effect size Cohen’s *d* to be 0.84 $\left( {\frac{t}{\sqrt n }} \right)$ for the effect of paired *t*-tests in our experimental design. The suggested sample size was approximately 16 to obtain at least 95% power for the effect of paired *t*-test in the overall mean condition at a significance level of 0.05. Eighteen participants were recruited in Experiment 1 to ensure adequate power.

Before participation, all individuals provided signed informed consent. The study received approval from the Research Ethics Board of Sun Yat-sen University and was conducted in accordance with the approved guidelines.

#### Stimuli and apparatus

The experiment was run on a 27-inch LCD monitor, positioned at a viewing distance of 57 cm, with a resolution of 2560 × 1440 pixels and a refresh rate of 60-Hz. The background was black (0, 0, and 0; RGB). The experiment was programmed using MATLAB (MathWorks, Natick, MA, USA) with Psychtoolbox extensions (Brainard, 1997; Pelli, 1997).

**Fig. 2:**
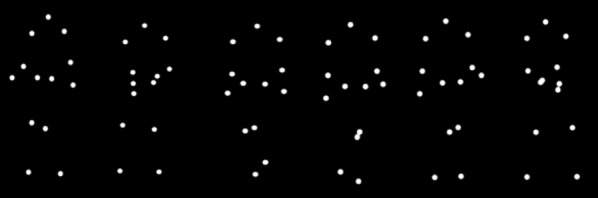
Stimuli. From left to right, the walking directions were − 40°, − 30°, − 20°, 20°, 30°, and 40°

Point light displays (PLDs) were used to represent walking (Fig. 2). They were selected from the Motion Capture Database (http://mocap.cs.cmu.edu) built by the Graphics Lab at Carnegie Mellon University. This database offers a diverse array of PLDs, consisting of 13 points of light with 60 frames/s. The distribution of these 13 points was located in the following locations on the body: one on the head, two on the shoulders, two on the elbows, two on the wrists, two on the hips, two on the knees, and two on the ankles. We chose a sequence with walking movement from the database as our experimental stimulus. Every animation consisted of 30 distinct frames and was displayed in loop.

The memory display consisted of eight BM with different walking directions, four on the left side and four on the right side of the screen, constituting two subsets. The four BM of each subset were randomly distributed in a 2 × 2 invisible square (each square: 3.89° × 3.89°), positioned 5.56° to the left or the right of the center of the screen. Each BM randomly deviated 0° to 0.56° from the center of the square in which it was placed. The probe (2.23° × 2.23°) appeared in the center of the screen.

There were six levels of the mean directions of each subset (− 40°, − 30°, − 20°, 20°, 30° and 40°). The mean directions of both subsets must be one positive and the other negative, and the combination of 20° and − 20° was excluded to avoid the two subsets being too similar, resulting in 8 combinations of subset means. For each combination of subset means, the left and right positions of each subset were counterbalanced. The four BM in each subset were derived from the subset’s mean (plus − 15°, − 5°, 5°, and 15°). Probe directions were determined by the mean (the mean of the left subset/the mean of the right subset) plus an angle ranging from − 30° to 30° in 5° increments.

#### Experiment design and procedure

**Fig. 3:**
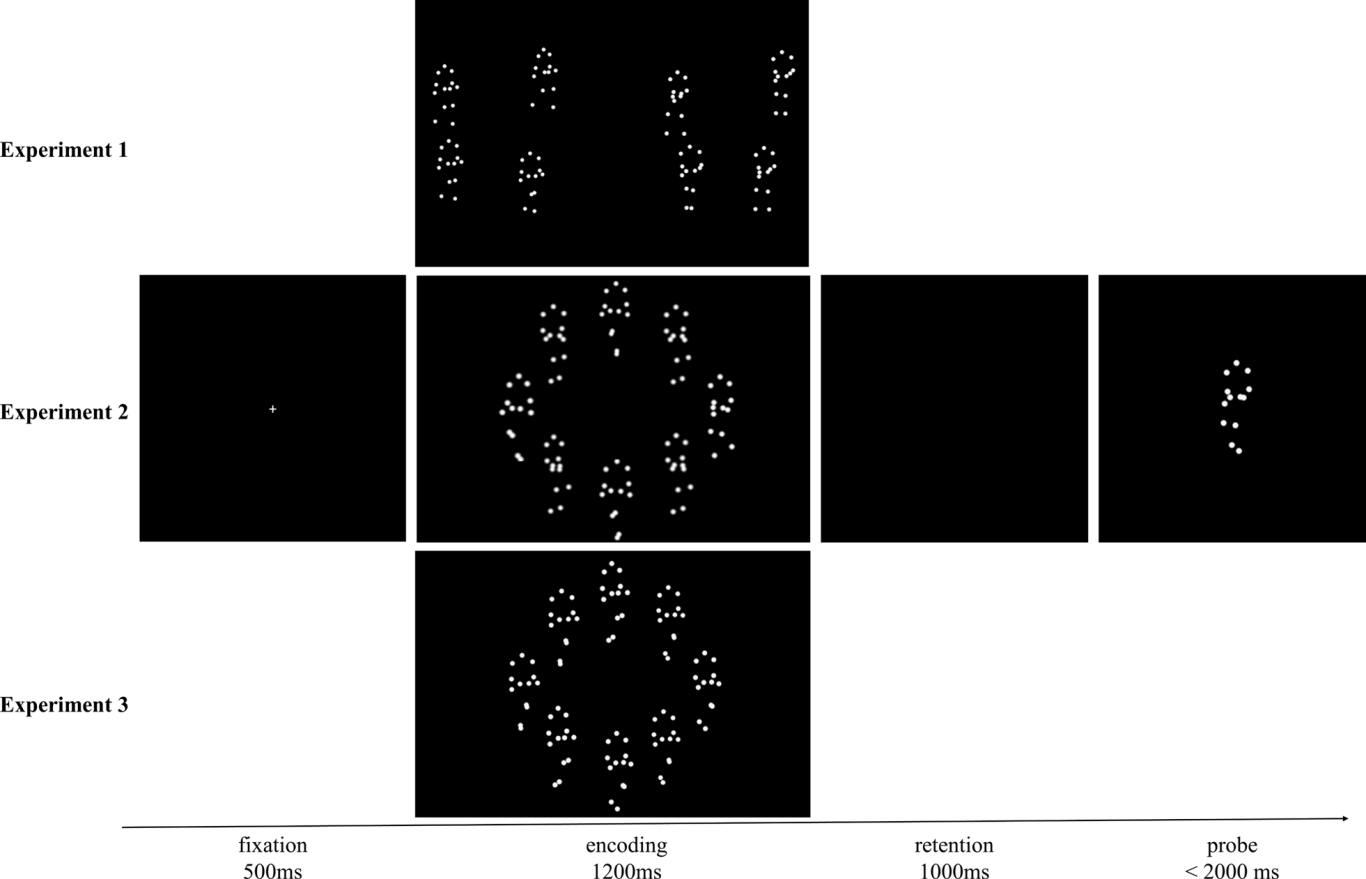
Procedure illustration in Experiments 1–3. Participants were required to remember the walking directions of all presented stimuli first. After a 1000-ms blank interval, a probe appeared. Participants should judge whether it was the member of the previous memory display

The experimental procedure is illustrated in Fig. 3. After a 500 ms fixation, the memory display composed of eight BM was presented for 1200 ms, which participants were required to all memorized. Then, a blank retention interval lasted for 1000 ms. Finally, a probe appeared in the center of screen. Participants should judge whether it was the member of the previous memory display (“Y” for “yes,” “N” for “no”). Responses should be completed within 2000 ms.

The experiment used a 2 (probe type: the left subset condition/the right subset condition) × 13 (probe offset: -30° to 30° in 5° increments) within-subjects design. The first factor indicated the mean in reference to which the probe offset was calculated. Probe offset represents the orientation difference between the according mean direction and the probe. Each combined condition contained 16 trials, which were randomly divided into four blocks. Before formal trials, 16 practice trials were required to ensure that participants understood the procedure. The entire task took approximately 50 min.

#### Data analysis

Only trials with reaction time (RT) longer than 200 ms and shorter than 2000 ms were included in further analysis. To detect whether the ensemble representations of subsets were represented, we merged trials in the left subset condition and the right subset condition and took the absolute values of probe offsets under these two conditions. The proportions of ‘yes’ responses were initially subjected to a one-factor seven-level (the absolute values of probe offset) repeated measures of variance (ANOVA). Then, we conducted separate comparisons between the proportion of ‘yes’ responses at each offset and that at the mean direction through paired *t*-tests to examine the extent to which the group-based ensembles were represented. Finally, the proportion of ‘yes’ responses at the mean direction was compared to the proportion combined across all memory directions using a paired *t*-test to directly test the group-based ensemble hypothesis.

### Results and discussion

**Fig. 4:**
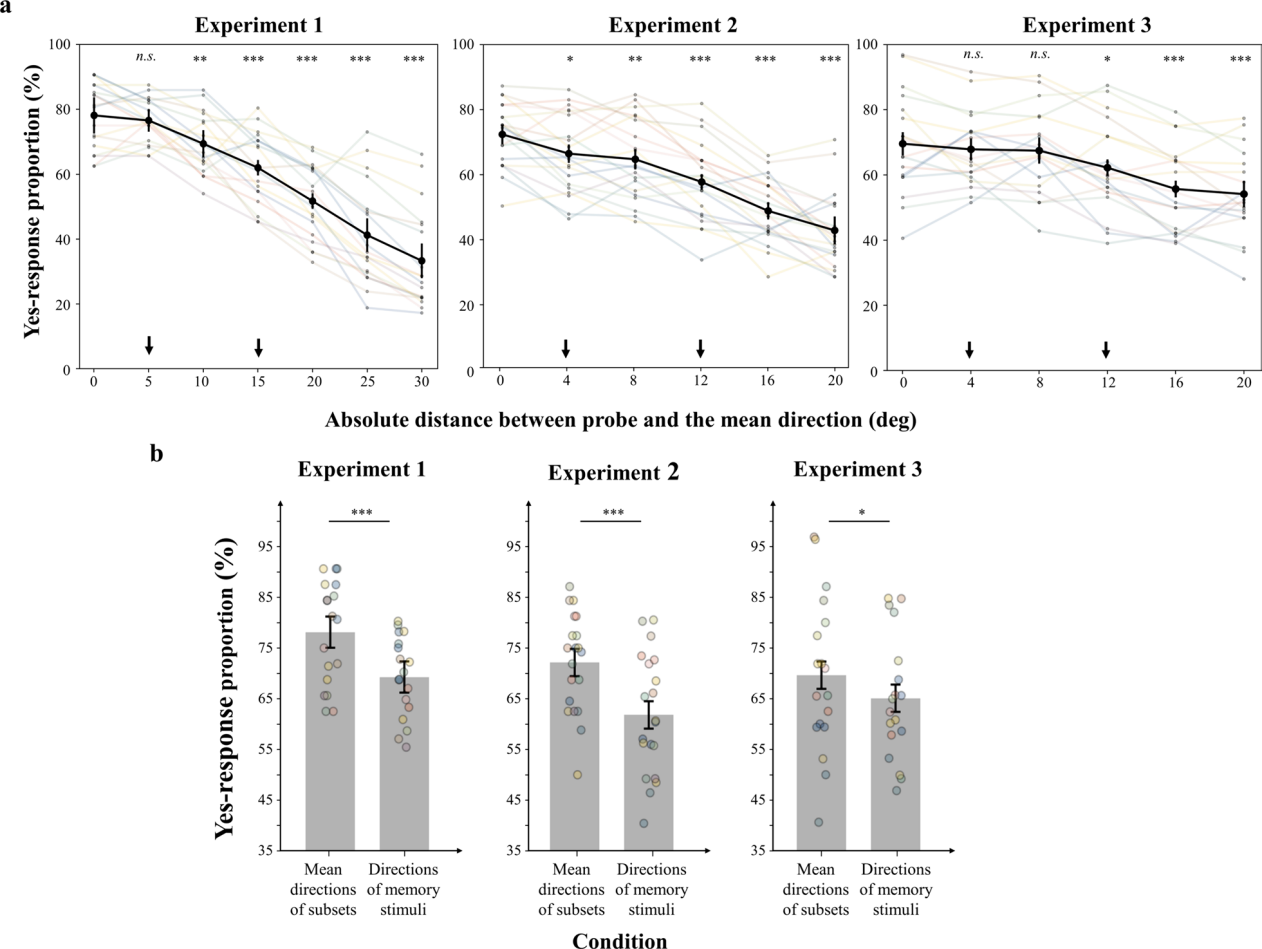
Results in Experiments 1–3. **a** The x-axis represents the absolute distance between probe orientation and the mean direction of subsets, and the y-axis shows the proportion of “yes” responses. The black dots represented group mean, with error bars indicating the within subject 95% confidence intervals. The smaller colorful dots and lines represented the results of each participant. The directions indicated by the arrows were the directions of the memory stimuli. ‘*’ represented the significance of the data comparing to proportion at the 0° offset. n.s. > 0.05, *p < 0.05, **p < 0.005, ***p < 0.001. **b** The proportion of ‘yes’ responses was plotted as a function of the type of the probe in Experiments 1–3 separately. The bars represented group mean, with error bars indicating the within subject 95% confidence intervals. The smaller colorful dots represented the results of each participant. *p < 0.05, ***p < .001

5.6% of trials were removed for abnormal RTs. The ANOVA revealed a significant main effect of probe offset [*F*(1, 6) = 73.93, *p* < 0.001, η_*p*_^2^ = 0.813]. And a sharp tuning of ‘yes’ responses around the mean of subsets (probe offset = 0°) appeared (Fig. 4a). Eleven out of eighteen participants had the highest proportion of ‘yes’ responses to the BM with a walking direction of the mean direction of subsets. These results suggest that the mean directions of subsets were maintained during the delay. Specifically, after correcting for multiple comparisons, the proportion of ‘yes’ responses was significantly higher when the probe offset was 0° compared to the range of 10–30° [5°: 1.6%, *t*(17) = 0.951, *p* = 0.355, Cohen’s *d* = 0.22, 95%CI for mean difference = [− 4.3%, 7.4%]; 10°: 8.8%, *t*(17) = 4.095, *p* = 0.002, Cohen’s *d* = 0.97, 95%CI for mean difference = [1.1%, 16.4%]; 15°: 16.1%, *t*(17) = 5.650, *p* < 0.001, Cohen’s *d* = 1.33, 95%CI for mean difference = [6.0%, 26.3%]; 20°: 26.4%, *t*(17) = 9.025, *p* < 0.001, Cohen’s *d* = 2.13, 95%CI for mean difference = [16.0%, 36.9%]; 25°: 36.9%, *t*(17) = 8.009, *p* < 0.001, Cohen’s *d* = 1.89, 95%CI for mean difference = [20.5%, 53.4%]; 30°: 44.8%, *t*(17) = 9.711, *p* < 0.001, Cohen’s *d* = 2.29, 95%CI for mean difference = [28.4%, 61.3%]] (Fig. 4a). Further paired-*t* tests showed that the mean proportions of ‘yes’ responses at the mean directions of subsets were even higher than that for the directions of the memory display [5° and 15°; 8.8%; *t*(17) = 4.293, *p* < 0.001, Cohen’s *d* = 1.01, 95%CI for mean difference = [4.5%, 13.2%] (Fig. 4b). Collectively, these results supported the group-based ensemble hypothesis.

**Fig. 5:**
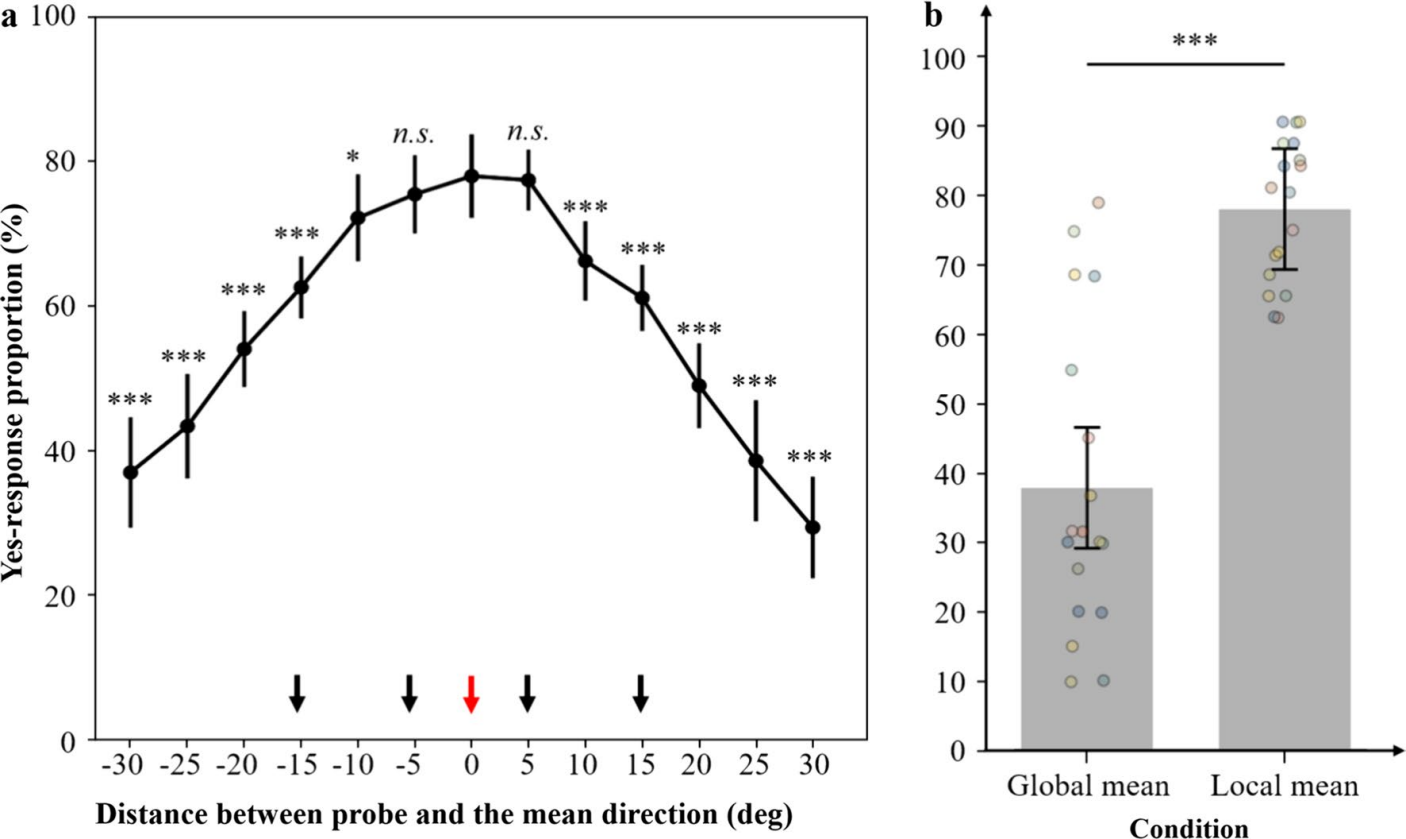
Results in Experiment 1. **a** The x-axis represents the distance between probe orientation and the mean direction of subsets, and the y-axis shows the proportion of “yes” responses. The black dots represented group mean, with error bars indicating the within subject 95% confidence intervals. The directions indicated by the black arrows were the directions of the memory stimuli. The direction indicated by the red arrow was the directions of the subset mean. ‘*’ represented the significance of the data comparing to proportion at the 0° offset. n.s. > 0.05, *p < 0.05, ***p < 0.001 (for detailed statistical results, see Table S1 in Appendix). **b** The proportion of ‘yes’ responses was plotted as a function of the type of the probe. The bars represented group mean, with error bars indicating the within subject 95% confidence intervals. ***p < 0.001

However, a potential limitation exists that the proportion of “yes” responses to the BM with a walking direction of the overall mean was higher compared to the subset mean. To rule out this possibility, we further obtained the function representing the proportion of “yes” response in relation to the probe offset relative to the subset mean. In Experiment 1, the probes with negative probe offsets, referenced to the positive subset mean, were closer to the global mean. Conversely, the probes with positive probe offsets, referenced to the negative subset mean, were closer to the global mean. Therefore, we first inverted the sign of the probe offsets determined relative to the negative subset mean. Consequently, the probes with negative offsets were consistently closer to the global mean, and the probes with positive offsets were farther, regardless of the subset mean used for reference. Then, we calculated the proportion of “yes” response for each probe offset relative to the local mean. While the curves of the proportions varying with the offset display a slight bias toward the global mean (the negative direction), the proportion at the subset mean remains the highest (Fig. 5a,). Moreover, we further conducted a paired-*t* test to directly compare the proportion at the global mean with that at the local mean (Fig. 5b). The results revealed a significant lower proportion of “yes” responses for the global mean compared to the local mean [− 40.2%, *t*(17) = − 6.882, *p* < 0.001, Cohen’s *d* = − 1.62, 95%CI for mean difference = [− 52.5%, − 27.9%]. Taken together, these findings provided additional evidence for that ensemble representations of the subsets were primarily maintained within the context of scenario structured by proximity.

## Experiment 2: WM representations for BM crowds structured by similarity

### Methods

In Experiment 2, we advanced Experiment 1 by removing the spatial proximity cues to examine the sole effects of directional similarity. The sample size was determined in the same way as Experiment 1. A separate group of 20 participants were recruited. The experimental procedure was similar to Experiment 1. The only two differences were that the memory stimuli were presented uniformly on an invisible circle (diameter = 8.89°) in the center of the screen (Fig. 3). To make the memory stimuli within each subset more similar to each other, their offsets from the means of the subsets were reduced to − 12°, − 4°, 4°, and 12°. Accordingly, the probe offsets were reduced to the range of − 20–20° in 4° increments. Other experimental settings were the same as Experiment 1.

### Results and discussion

2.9% of trials were removed for abnormal RTs. The ANOVA revealed a significant main effect of probe offset [*F*(1, 5) = 57.45, *p* < 0.001, η_*p*_^2^ = 0.751]. And a sharp tuning of ‘yes’ responses around the mean directions of the subsets (probe offset = 0°) appeared (Fig. 4a). Thirteen out of twenty participants had the highest proportion of ‘yes’ responses to the BM with the walking direction of the mean directions of subsets. These results suggest that the mean directions of subsets were indeed represented during the delay. Specifically, after correcting for multiple comparisons, the proportion of ‘yes’ responses was significantly higher when the probe offset was 0° compared to other offsets [4°: 6.0%, *t*(19) = 3.015, *p* = 0.021, Cohen’s *d* = 0.67, 95%CI for mean difference = [− 0.7%, 12.6%]; 8°: 7.7%, *t*(19) = 3.966, *p* = 0.003, Cohen’s *d* = 0.89, 95%CI for mean difference = [1.2%, 14.2%]; 12°: 14.7%, *t*(19) = 7.518, *p* < 0.001, Cohen’s *d* = 1.68, 95%CI for mean difference = [8.1%, 21.3%]; 16°: 23.6%, *t*(19) = 11.651, *p* < 0.001, Cohen’s *d* = 2.61, 95%CI for mean difference = [16.8%, 30.4%]; 20°: 29.7%, *t*(19) = 12.303, *p* < 0.001, Cohen’s *d* = 2.75, 95%CI for mean difference = [21.6%, 37.8%]] (Fig. 4a). Further paired-*t* tests showed that the average proportions of ‘yes’ responses to the mean directions of subsets were even higher than that for all directions of the memory display [4° and 12°; 10.3%; *t*(19) = 5.67, *p* < 0.001, Cohen’s *d* = 1.27, 95%CI for mean difference = [6.5%, 14.1%]] (Fig. 4b). Consistent with Experiment 1, the group-based ensemble hypothesis was supported.

**Fig. 6:**
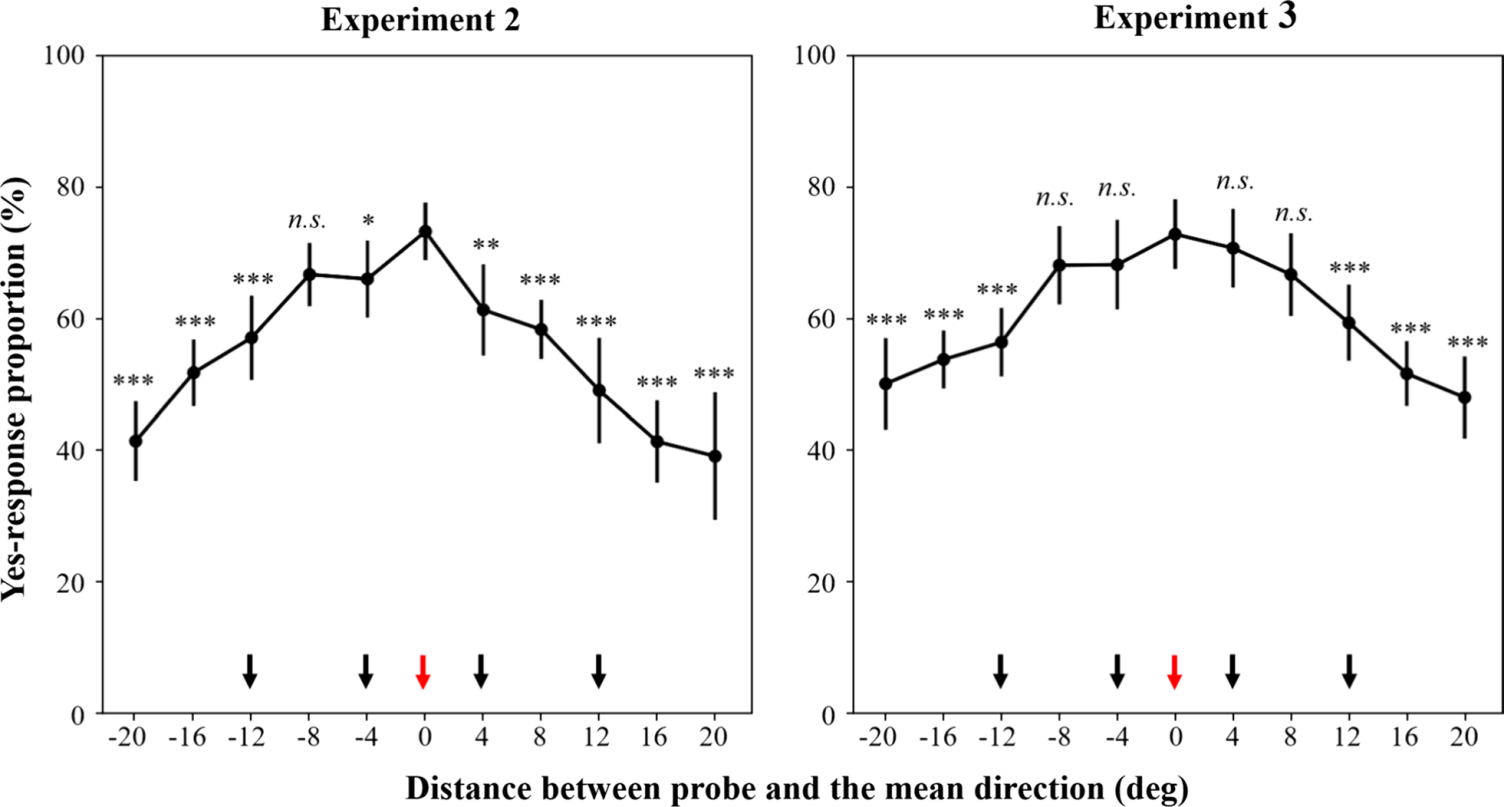
Results in Experiments 2 and 3. The x-axis represents the distance between probe orientation and the mean direction of subsets, and the y-axis shows the proportion of “yes” responses. The black dots represented group mean, with error bars indicating the within subject 95% confidence intervals. The directions indicated by the black arrows were the directions of the memory stimuli. The direction indicated by the red arrow was the directions of the subset mean. ‘*’ represented the significance of the data comparing to proportion at the 0° offset. n.s. > 0.05, *p < 0.05, **p < 0.005, ***p < 0.001 (for detailed statistical results see Table S2 and Table S3 in Appendix)

Similar to Experiment 1, we also obtained the function representing the proportion of ‘presence’ response in relation to the probe offset relative to the subset mean. The curve also displayed a slight bias toward the global mean (the negative direction), but the proportion at the subset mean still remained the highest (Fig. 6). Due to the experimental settings, it is challenging to directly compare the proportions at the global mean with that at the local mean in Experiment 2. Nevertheless, this concern can be addressed in Experiment 3.

## Experiment 3: Reduction in the strength of similarity

### Methods

In Experiment 3, we ruled out the effects of directional consistency. The sample size was determined in the same way as Experiment 1. A separate group of 18 participants was recruited. The experimental procedure was similar to Experiment 2. The differences were as follows. The means of the subsets own the same symbol, i.e., both were positive or both were negative, leading the strength of the similarity cue to be reduced. And there were only two combinations of subset means (20° and 65°, − 20° and − 65°). The other experimental settings were the same as Experiment 2 (Fig. 3).

### Results and discussion

1.4% of trials were removed for abnormal RTs. The ANOVA revealed a significant main effect of probe offset [*F*(1, 5) = 16.880, *p* < 0.001, η_*p*_^2^ = 0.50]. And a sharp tuning of ‘yes’ responses around the mean directions of subsets (probe offset = 0°) appeared (Fig. 4a). Nine out of eighteen participants had the highest proportion of ‘yes’ responses to the mean directions of subsets. Specifically, after correcting for multiple comparisons, the proportion of ‘yes’ responses was significantly higher when the probe offset was 0° compared to the range of 12–20° [4°: 1.7%, *t*(19) = 0.806, *p* = 1.000, Cohen’s *d* = 0.19, 95%CI for mean difference = [− 5.6%, 9.0%]; 8°: 2.1%, *t*(17) = 0.745, *p* = 1.000, Cohen’s *d* = 0.18, 95%CI for mean difference = [-7.6%, 11.8%]; 12°: 7.3%, *t*(17) = 3.656, *p* = 0.015, Cohen’s *d* = 0.86, 95%CI for mean difference = [0.5%, 14.2%]; 16°: 13.9%, *t*(17) = 6.716, *p* < 0.001, Cohen’s *d* = 1.58, 95%CI for mean difference = [6.8%, 20.9%]; 20°: 15.4%, *t*(17) = 6.211, *p* < 0.001, Cohen’s *d* = 1.46, 95%CI for mean difference = [7.0%, 23.9%]] (Fig. 4a). Further paired-*t* tests showed that the average proportions of ‘yes’ responses to the mean directions of subsets were even higher than that for all directions of the memory display [4° and 12°; 4.5%; *t*(17) = 2.51, *p* = 0.022, Cohen’s *d* = 0.59, 95%CI for mean difference = [0.7%, 8.3%]] (Fig. 4b). Consistent with Experiments 1 and 2, the group-based ensemble hypothesis was supported, although the line of proportion decreased less steep, showing a less apparent effect than that in Experiments 1 and 2.

Similarly, we further obtained the function representing the proportion of ‘presence’ response in relation to the probe offset relative to the subset mean. The only difference was that we inverted the sign of the probe offsets determined relative to the smaller subset mean (25° or − 65°). The proportion at the subset mean remained the highest. Moreover, we further conducted a paired-*t* test to directly compare the proportion at the global mean with that at the local mean (Fig. 6). Although the global mean was not directly probed in Experiment 3, the distance between the global and local means was fixed at 22.5°. Therefore, the point at − 20° on the function representing the proportion of ‘presence’ response in relation to the probe offset relative to the subset mean was consistently 2.5° away from the global mean. This was considered a reasonable approximation of the point representing the global mean. Consequently, we compared the proportion at − 20° and that at the local mean in Experiment 3. The results showed that the proportion of ‘yes’ response for the global mean was significantly lower compared to the local mean [− 22.8%, *t*(17) =  − 4.586, *p* < 0.001, Cohen’s *d* = − 1.08, 95%CI for mean difference = [-33.3%, − 12.3%], indicating that WM primarily maintained the ensemble representations of the subsets within the context of scenario structured by similarity.

### General discussion

The current study uncovered how BM crowds characterized by hierarchical structures were stored in WM. To achieve this, we employed the member identification task as an implicit measure to probe the specific representations maintained during the delay. In Experiment 1, where BM crowds were spatially partitioned into two separate units, participants exhibited a tendency to represent organized units as distinct ensembles in WM. These results provide support for the group-based ensemble hypothesis. Furthermore, Experiment 2 demonstrated that participants automatically maintained group-based ensemble representations even when multiple BM grouped by similarity were uniformly distributed. This result indicates directional similarity alone is sufficient to trigger the formation of subgroup-based ensemble representations in WM. Experiment 3 further corroborated the previous observations by reducing the strength of the similarity cue. After setting the directions of all memory stimuli to the same symbol, the results remained the same, indicating that group-based ensemble representations were still formed despite the unobvious similarity cue. Taken together, these findings provide compelling evidence for the existence of group-based ensemble representations in WM encoding of BM crowds.

In Experiment 1, we employed proximity as the organizational cue. Previous research comparing the roles of spatial and non-spatial grouping has highlighted the beneficial impact of proximity on subjects' statistical representation accuracy (Im & Chong, 2014). However, in our study, although no direct comparison between experiments was conducted, the ensemble coding effect stemming from proximity grouping did not exhibit a discernible difference from similarity in terms of effect size. It is possible that the greater within-group difference in Experiment 1 offset any potential benefits derived from proximity. But this difference may also be attributed to differences in task requirements. Participants in Im’s study were explicitly required to form ensemble representations of each group, while in our study ensemble representation was tested implicitly. It is conceivable that only when the task explicitly demands participants to more accurately estimate, the group mean would they actively leverage proximity to enhance their estimations.

In contrast, in In Experiments 2 and 3, we employed similarity as the organizational cue. When similarity serves as the organizational cue, we found that, the distributional properties of each subgroup can significantly influence the grouping process. Recent findings by Ortego and Störmer (2024) demonstrate that participants' ensemble representations of one group tend to be biased toward the other group when the two groups overlap in feature space. In our study, we avoided overlap in the distribution of walking direction between two BM subsets, thus preserving the integrity of ensemble representations for each subset and mitigating any mutual influence between subsets. Moreover, researchers have proposed a series of factors affecting ensemble coding, including group differences in mean value and items’ distance to mean (Im et al., 2021; Treue et al., 2000; Utochkin, 2015). A population-coding model of ensemble perception has been proposed for these various facets of ensemble perception (Utochkin et al., 2024). While these studies mainly focused on the grouping of simple features, our study extends these insights to BM to some extent. Comparing Experiments 3 versus 2, we notice that a smaller group differences in mean value resulted in a weaker bias to the group’s mean, suggesting a less efficient ensemble coding due to diminished group disparity. However, it remains to be explored whether existing models accurately predict the grouping process of BM, warranting further investigation in this domain.

Although our results demonstrate that WM inherently organizes structured BM crowds into separate ensembles, the simultaneous maintenance of both ensemble representations for the two subsets in WM remains uncertain. Several studies have explored the feasibility of extracting and concurrently storing multiple ensemble representations in the context of ensemble perception, providing affirmative findings (Attarha & Moore, 2015; Attarha et al., 2014). However, this question persists within the framework of WM, which is characterized by limited capacity, particularly when dealing with complex features such as BM. This complexity is particularly pronounced given that while WM can typically store 3–4 simple features, the capacity for BM is confined to 2–3 instances (Gao et al., 2016a, 2016b; Wood, 2007). This disparity poses a challenge to maintaining numerous ensembles of BM within the WM framework. Future research could further investigate the simultaneous storage of ensemble representations for separate groups within WM.

Echoing previous studies which indicated that ensemble encoding was sensitive to grouping cues, our results provide direct evidence that WM efficiently groups information using Gestalt principles and simultaneously compresses this information as separate ensembles. Grouping and ensemble coding have both been extensively assumed as the strategies to alleviate capacity limitations (Alvarez, 2011; Ariely, 2001; Brady et al., 2009; Peterson et al., 2015; Xu & Chun, 2007). In light of this, the interaction between the two strategies has attracted intensive attention of studies, which confirmed the sensitivity of ensemble coding to grouping cues (Brady et al., 2011; Corbett, 2017; Lamer et al., 2018). For instance, researchers found that participants’ judgements of summary statistics were less accurate when stimuli contained grouping structures (Lew & Vul, 2015; Marchant et al., 2013). Advancing these results, the current study directly reveals the simultaneous operating mechanisms of these two strategies in WM, i.e., WM automatically represents environmental regularities as subgroup-based ensemble representations. Besides, implicit ensemble tasks were adopted in the current study in which participants don’t need directly report the mean of a stimuli set and were encouraged to memorize the individual stimulus, thus better verifying the robustness and automaticity of the pattern of subgroup-based ensemble representations in WM.

This study contributes to and extends research on ensemble coding, which has shown that BM can be automatically stored as ensembles in WM, in addition to simple features. There is substantial evidence that summary information, such as the mean, can be precisely and rapidly perceived from various types of features, including simple features (e.g., Alvarez, 2011; Michael et al., 2014) and complex social features (e.g., Elias et al., 2017; Florey et al., 2016; Yamanashi Leib et al., 2016), including the walking directions of BM (Sweeny et al., 2013). However, the question of whether ensembles can be maintained in WM has only been addressed for simple features (Brady & Alvarez, 2011; Brady & Alvarez, 2015; Brady et al., 2011; Schurgin & Brady, 2019). In this context, our findings fill this gap and provide further evidence for the generality of ensemble coding. Namely, in WM, a higher-level cognitive process, complex features such as BM can still be automatically represented as ensembles.

Our findings also present new evidence that representations in WM are not independent, but rather interconnected. Traditionally, WM has been conceived as a repository for storing limited and discrete items (Adam et al., 2017; Luck & Vogel, 1997; Zhang & Luck, 2008), often employing individual biological motion (BM) elements to explore WM capacity (Gao et al., 2016a, 2016b; Wood, 2007) and neural representations of BM (Cai et al., 2018; Gao et al., 2015). Nevertheless, recent years have witnessed a growing body of evidence challenging this classical notion, with studies revealing interactions between WM representations (Bae & Luck, 2017; Czoschke et al., 2020; Lively et al., 2021; Utochkin & Brady, 2020). This shift in understanding is also mirrored in BM studies. For instance, Ding et al. (2017) observed that interactive BM was stored in WM as a unified chunk, yielding better memory performance compared to individual memorization. Consistent with these results, our study showed that participants inherently uphold ensemble representations, rather than isolated individual BM representations, within WM.

Although our previous discussion primarily focused the ensemble representations of BM within WM, we do not deny the existence of at least some memories for individual BM. In fact, drawing upon the hierarchical encoding theory, we posit that individual memories exist in the current study. Based on this theory, WM encodes both the “gist” of WM displays (ensemble statistics such as mean value) and information about specific items (Brady & Alvarez, 2011). Empirical evidence was largely derived from the delayed estimation task, wherein participants are tasked with reproducing a cued item from the memory display. The results of such studies have consistently revealed that the memories for individual items exhibit a bias toward the mean value, but displayed significantly lower errors compared to the chance level (Corbett, 2017; Duffy et al., 2010; Griffiths et al., 2018; Son et al., 2020). This suggests that WM retains information about individual items, and simultaneously, these individual representations are influenced by higher-order statistics (mean value) held in WM. Similarly, the member identification task employed in our current study also required the encoding of individual item memories. Therefore, we posit that some memories for individual items exist in the context of the current study.

Beyond the theoretical significance, this investigation provides valuable insights into the mechanisms and strategies employed by the human brain in processing and interpreting these social scenes, with practical implications across various domains. Firstly, understanding how individuals perceive and extract meaningful information from structured BM crowds can contribute to the development of effective crowd management strategies. For instance, our findings can inform the design of signage or visual cues that enhance safety in crowded environments such as airports or public gatherings. Additionally, our research has direct relevance to the domain of social interaction. The processing of structured BM crowds plays a crucial role in social contexts, where individuals rely on the interpretation of clustering structures to understand intentions and emotions. By gaining a better understanding of how people perceive these structures within crowds, we can improve social interaction in various settings. Finally, our research contributes to the development of computational models or algorithms that simulate human visual processing, advancing computer vision applications.

Finally, this study has some limitations. While constructing structured BM crowds, we employed fundamental organizational cues of proximity and similarity, both rooted in basic physical attributes. However, one of the distinguishing aspects of BM lies in their inherent social nature. Consequently, in addition to the application of Gestalt principles, there exist other distinctive social organizational principles pertinent to BM, such as joint gaze (Corkum & Moore, 1995). Gaze cues represent ubiquitous social features that furnish a wealth of information during social interactions, particularly concerning attention and intention (Emery, 2000; Moll & Tomasello, 2007). Future research endeavors could consider incorporating social cues like joint gaze to formulate structured BM crowds. This avenue of investigation would provide an opportunity to explore whether higher-level cognitive processes influence the encoding and storage of these BM crowds within WM.

### Supplementary Information

Supplementary material 1.

## Data Availability

Experimental data in an aggregated format and all materials are available on Open Science Framework https://osf.io/ezk4v/.

## Code availability

The experiment was programmed using MATLAB (MathWorks, Natick, MA, USA) with Psychtoolbox extensions (Brainard, 1997; Pelli, 1997).
