# Supplementary material for: The combination operation of grouping and ensemble coding for structured biological motion crowds in working memory
Source: Cogn Res Princ Implic. 2024 Jul 10;9:45. doi: 10.1186/s41235-024-00574-6 (PMC11236836; doi:10.1186/s41235-024-00574-6)
Supplement: Supplementary file 1 — Supplementary material 1. [file 41235_2024_574_MOESM1_ESM.docx]

**Appendix**


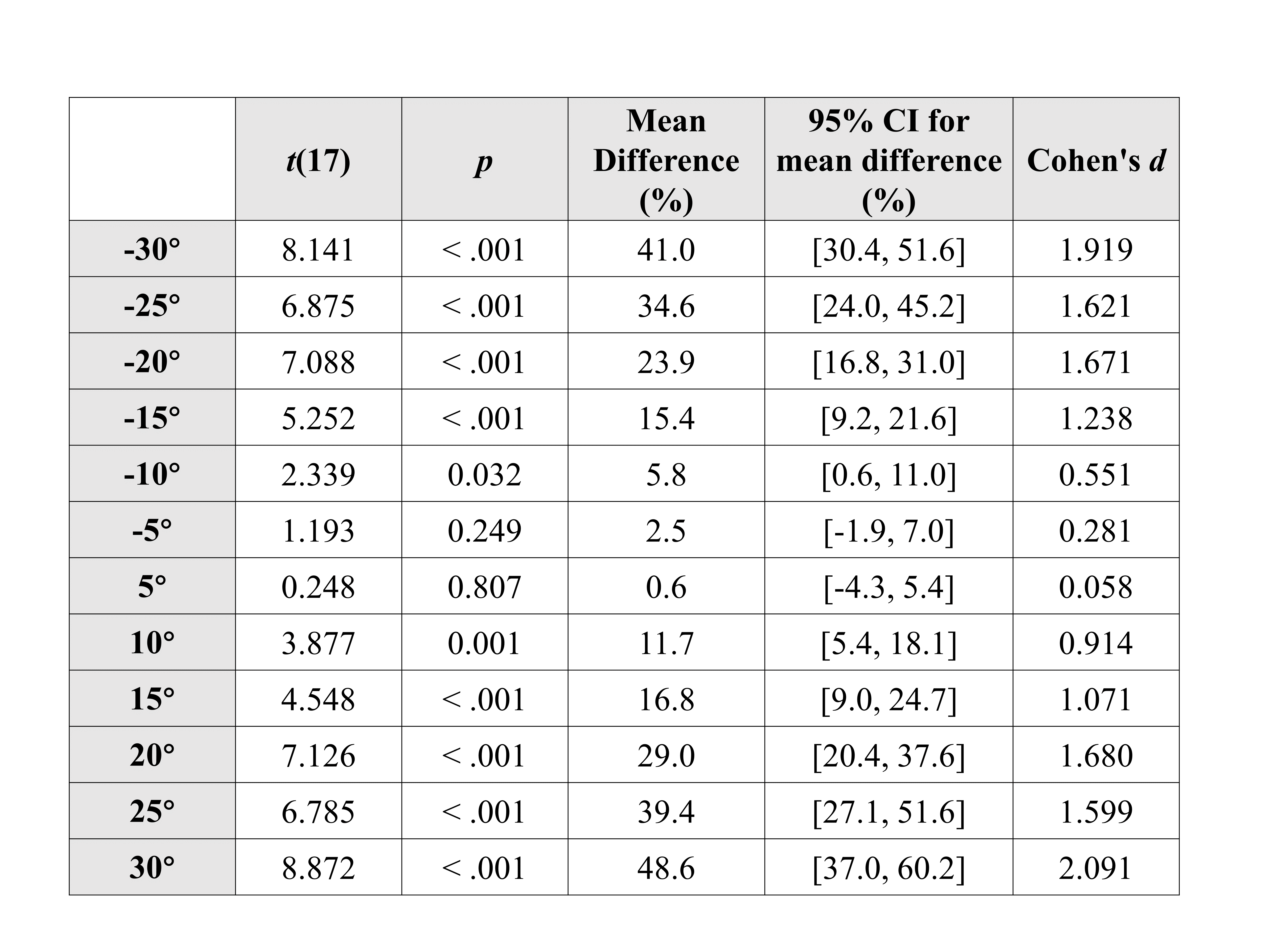


**Table S1**

*The supplementary results in Experiment 1.* Each row represented the significance of the proportion at the corresponding offset comparing to the proportion at the 0° offset.


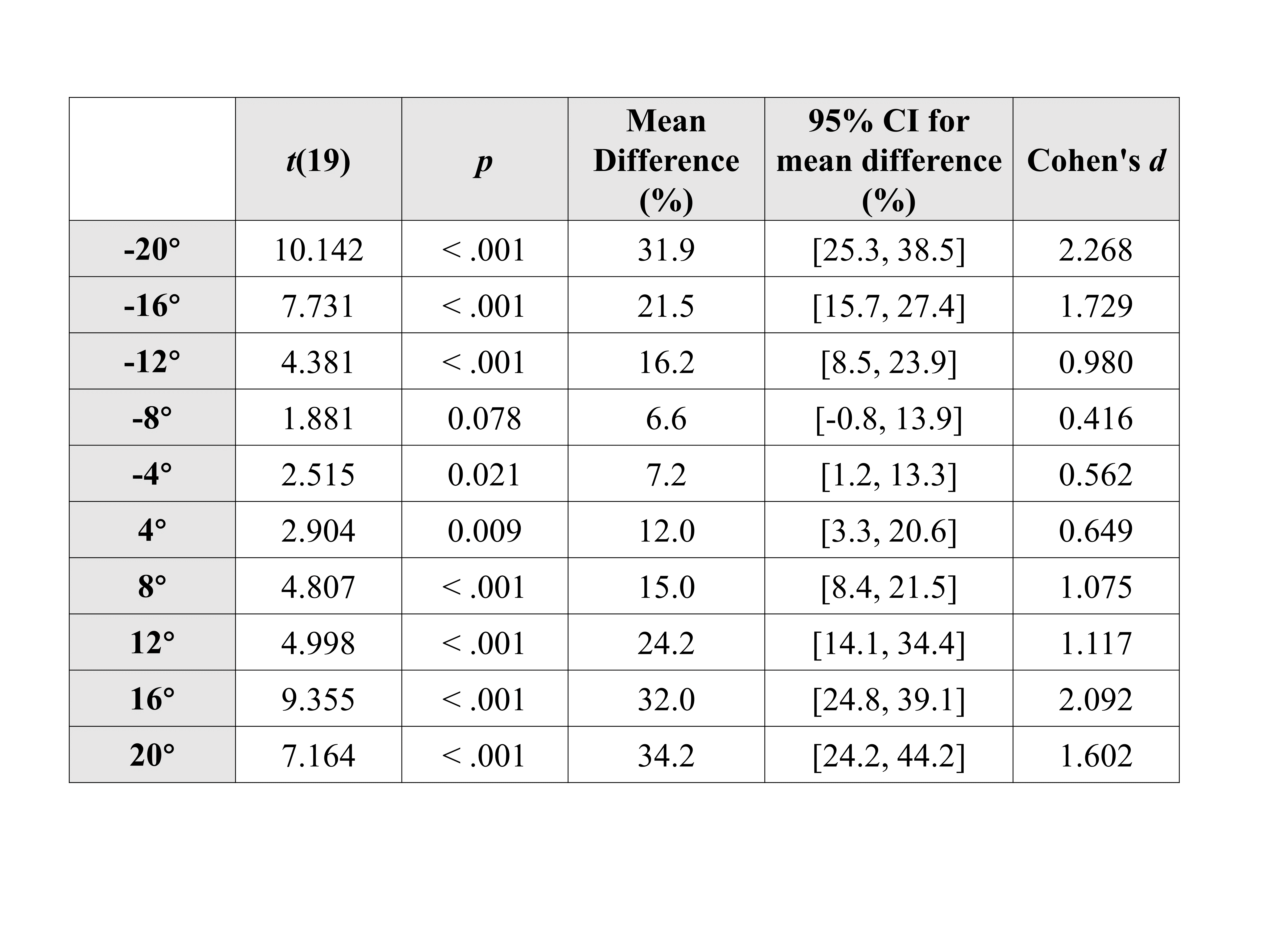


**Table S2**

*The supplementary results in Experiment 2.* Each row represented the significance of the proportion at the corresponding offset comparing to the proportion at the 0° offset.


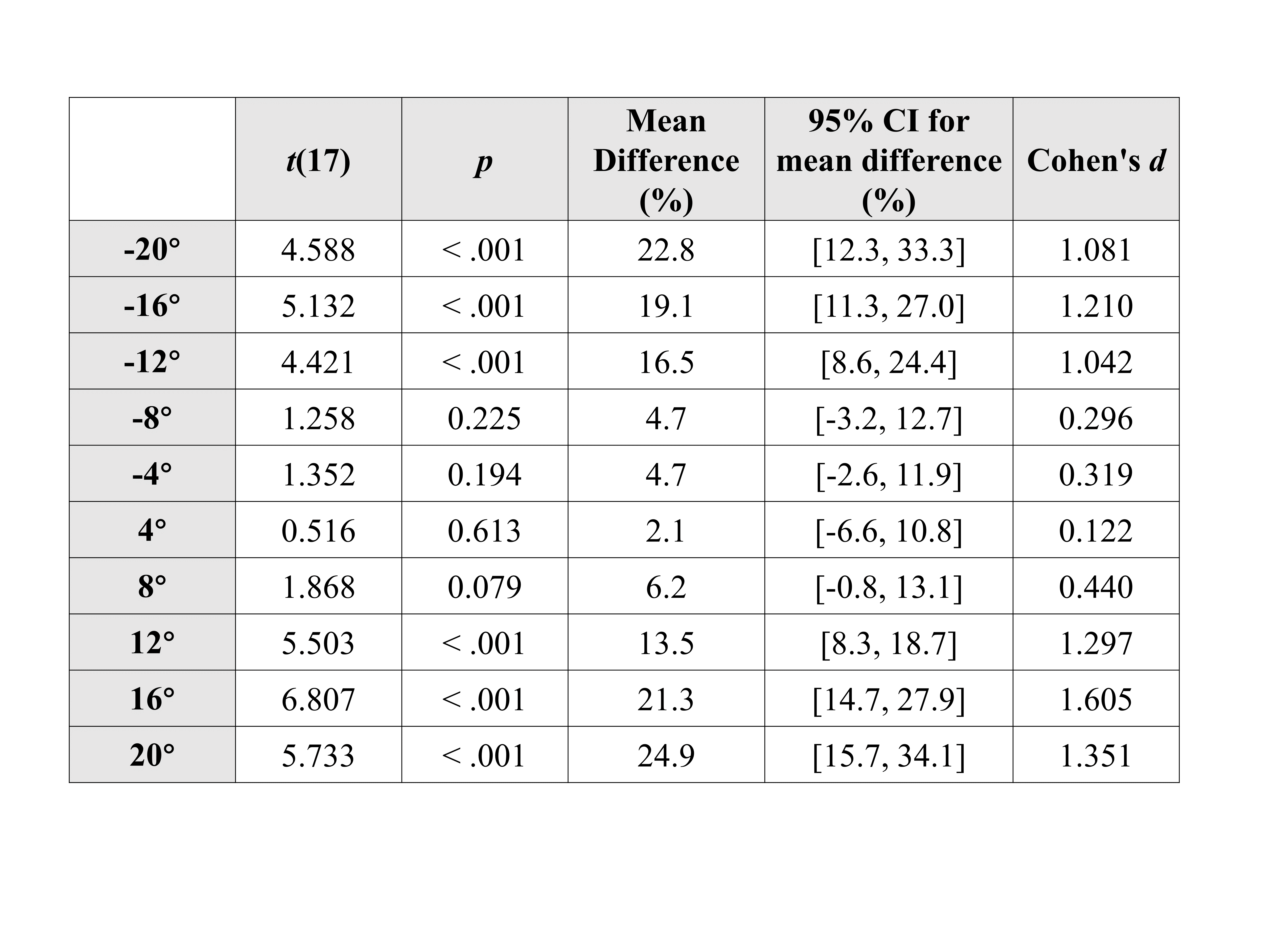


**Table S3**

*The supplementary results in Experiment 3.* Each row represented the significance of the proportion at the corresponding offset comparing to the proportion at the 0° offset.
